# Identifying recovery trajectories following primary total shoulder arthroplasty: a cohort study of 3,358 patients from the Dutch Arthroplasty Register

**DOI:** 10.2340/17453674.2025.43085

**Published:** 2025-03-04

**Authors:** Mirthe H W VAN VEGHEL, Liza N VAN STEENBERGEN, Cornelis P J VISSER, B Willem SCHREURS, Gerjon HANNINK

**Affiliations:** 1 Department of Orthopaedics, Radboud University Medical Center, Nijmegen; 2 Dutch Arthroplasty Register (Landelijke Registratie Orthopedische Interventies), ‘s-Hertogenbosch; 3 Department of Orthopaedic Surgery, Alrijne Hospital, Leiderdorp; 4 Department of Medical Imaging, Radboud University Medical Center, Nijmegen, the Netherlands

## Abstract

**Background and purpose:**

Some patients do not improve after total shoulder arthroplasty (TSA), indicating different recovery trajectories. We aimed to identify recovery trajectories after TSA based on the Oxford Shoulder Score (OSS). Second, we investigated whether recovery trajectories were associated with patient or procedure characteristics.

**Methods:**

We included primary anatomical and reversed TSAs (ATSAs/RTSAs) for osteoarthritis (OA) or cuff arthropathy/rupture with preoperative, 3-month, and/or 12-month postoperative OSS, registered between 2016 and 2022 in the Dutch Arthroplasty Register (n = 3,358). We used latent class growth modeling (LCGM) to identify recovery patterns, and multinomial logistic regression analyses to investigate associations between potential risk factors and class membership (odds ratio [OR], 95% confidence interval [CI]).

**Results:**

We identified 3 recovery patterns: “Fast responders” (59%), “Steady responders” (27%), and “Poor responders” (14%). Factors associated with “Steady responders” vs “Fast responders” were female vs male sex (OR 2.0, CI 1.5–2.7), ASA III–IV vs ASA I (OR 1.9, CI 1.2–3.1), Walch A1 vs B2 (OR 1.6, CI 1.1–2.5), and most vs medium socioeconomic deprivation (OR 1.4, CI 1.1–1.9). Factors associated with “Poor responders” vs “Fast responders” were ASA II vs ASA I (OR 2.0, CI 1.1–3.6), ASA III–IV vs ASA I (OR 3.0, CI 1.6–5.5), Walch A1 vs B2 (OR 2.1, CI 1.3–3.3), previous shoulder surgeries (OR 1.8, CI 1.3–2.4), most vs medium socioeconomic deprivation (OR 1.5, CI 1.2–2.0), RTSA for OA vs ATSA for OA (OR 1.8, CI 1.2–2.7), and RTSA for cuff arthropathy or rupture vs ATSA for OA (OR 2.3, CI 1.5–3.4).

**Conclusion:**

3 recovery trajectories were identified following TSA, which we labelled as “Fast responders,” “Steady responders,” and “Poor responders.” “Steady responders” and “Poor responders” were more likely to have higher ASA scores, a Walch A1 vs B2 classification, and greater vs medium socioeconomic deprivation than “Fast responders.” Moreover, “Steady responders” were more likely to be female, while “Poor responders” were more likely to have previous shoulder surgeries and RTSA for OA or for cuff arthropathy or rupture than “Fast responders.”

Although total shoulder arthroplasty (TSA) usually leads to substantial improvements in both pain and physical functioning, certain patients do not experience improvement or continue to report persistent pain 1 year after the procedure [1-3]. This indicates different recovery trajectories after TSA. However, little is known about the recovery trajectories after TSA. Gaining insight into the different recovery trajectories following TSA, as well as the patient and procedure characteristics associated with them, may provide valuable guidance for clinical decision-making [4].

Latent class growth modeling (LCGM) is an effective statistical technique for understanding heterogeneous recovery trajectories, as it enables the identification of different patient groups based on shared recovery patterns, rather than predefined patient categories [5]. While previous studies from the Dutch Arthroplasty Register (LROI) have successfully identified several recovery trajectories of patient-reported outcomes after total hip and knee arthroplasty using LCGM, recovery trajectories after TSA have not previously been identified with LROI data [6-8].

An earlier single-center study including small numbers of TSAs identified 3 recovery trajectories, which were named “High performers,” “Steady progressors,” and “Resistant responders” [9]. The study may have limited generalizability. Therefore, we aimed to identify recovery trajectories after TSA according to the Oxford Shoulder Score (OSS), using data from the national database, LROI. Second, we aimed to investigate whether recovery trajectories were associated with patient or procedure characteristics.

## Methods

### Data source

Data was obtained from the LROI, which is the national population-based arthroplasty register of the Netherlands. The completeness of primary shoulder arthroplasties is reported to be higher than 95% [10]. The LROI contains data on patient, prosthesis, and procedure characteristics of primary and revision arthroplasties as well as patient-reported outcome measurements (PROMs). PROMs after TSA have been registered in the LROI since 2016 and include the EuroQol 5 Dimensions index score (EQ-5D index), the EuroQol Visual Analog Scale (EQ VAS), the OSS, and the Numeric Rating Scale during activity (NRS activity) and at rest (NRS rest). These PROMs are measured preoperatively (within 182 days before surgery), and at 3 months (between 63 and 110 days) and 12 months (between 323 and 407 days) postoperatively.

This study was reported in accordance with the STROBE guidelines.

### Participants

**Figure 1:**
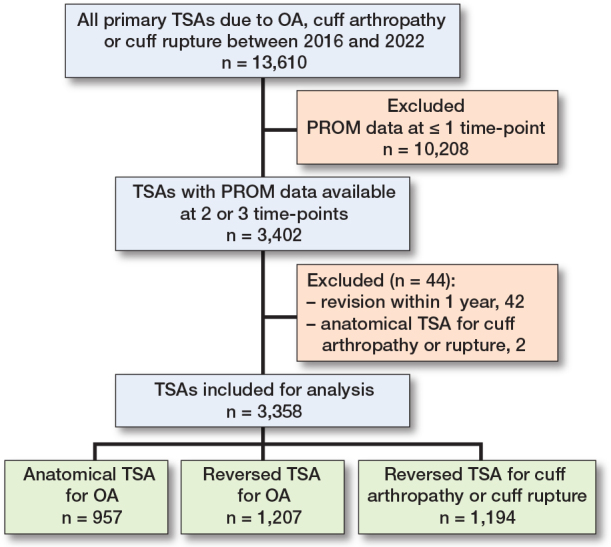
Flowchart. TSA = total shoulder arthroplasty; OA = osteoarthritis; PROM = patient-reported outcome measurement.

We included all primary anatomical TSAs (ATSAs) and reversed TSAs (RTSAs) due to glenohumeral osteoarthritis (OA), cuff arthropathy, or cuff rupture registered in the LROI between 2016 and 2022, with PROM data available at least at 2 of the 3 time-points (n = 3,358; Figure 1). The LROI defines cuff arthropathy as “osteoarthritis of the shoulder joint as a consequence of the tendons around the shoulder joint being affected,” and cuff rupture as “rupture of a tendon of the muscles that are around the shoulder joint” [10]. TSAs that were revised within 1 year (n = 42) were excluded. The number of ATSAs for cuff arthropathy or rupture (n = 2) was small, and these cases were therefore excluded as well.

PROM responders and non-responders were similar in terms of sex, age, diagnosis and arthroplasty type, American Society of Anesthesiologists (ASA) class, body mass index (BMI), Walch classification, smoking status, previous surgeries on the affected shoulder, socioeconomic status (SES), surgical approach, and fixation method. Therefore, we considered the missing cases to be missing at random. Non-responders were more frequently treated in the early years of the study (i.e., 2016–2017) and were therefore more often operated on using the anterosuperior or superolateral approach than responders, as this approach was more commonly used in the early years of our study.

### Outcome

The primary outcome was the recovery trajectory, based on patient-reported pain and physical function, as measured by OSS. The OSS was chosen as it is the only shoulder-specific PROM available in the LROI. The OSS is a validated PROM designed to evaluate shoulder surgery outcomes. It consists of 12 questions assessing various aspects of shoulder function (e.g., dressing, shopping) and pain (e.g., worst pain, usual pain) experienced over the past 4 weeks. Each question is scored on a scale from 0 to 4, with 4 representing the best outcome. The total score ranges from 0 to 48, with higher values indicating better shoulder function and less pain [11]. Incomplete questionnaires are treated as missing data in the LROI database, accounting for only 1.1% of the missing preoperative OSS in the total population of 13,610 cases in our study. For the 3-month and 12-month postoperative OSS, the proportions were 0.7% and 0.8%, respectively.

### Statistics

Descriptive statistics were used to summarize patient and procedure characteristics. Diagnosis and type of shoulder arthroplasty were combined into a single variable with 3 categories: ATSA for OA, RTSA for OA, and RTSA for cuff arthropathy or rupture. Missing data ranged between 0.0% for sex and age, and 17% for 12-month postoperative OSS (Table 1). Missing data was imputed, using multiple imputation by chained equations using predictive mean matching in which 10 datasets were created. All estimates were pooled according to Rubin’s Rules. Subsequently, LCGM was performed using R (version 4.4.0, R Foundation for Statistical Computing, Vienna, Austria) with the “lcmm” package to identify possible subgroups of patients based on their recovery trajectories. A 1-class to 4-class latent class growth model was conducted. A model with additional classes was not clinically relevant, since only minor variations in the same recovery trajectories were observed. Model selection was determined by visually examining the plots, taking into account interpretability and clinical relevance, and evaluating the relative fit statistics Akaike Information Criterion (AIC), Bayesian Information Criterion (BIC) and Adjusted BIC of the model [12]. The final model was selected for having the lowest relative fit statistics, while still offering satisfactory interpretability and clinical relevance. Our method assumes that the response variables within each latent class are normally distributed. Violations of the normality assumption can affect the estimation of the latent classes, potentially leading to biased class assignments or incorrect inferences regarding the number of latent classes. However, the method is generally robust to moderate deviations from normality, particularly when sample sizes are large. Therefore, we performed diagnostic checks such as visual inspection of histograms and Q–Q plots for the residuals of each latent class, and distribution assumptions appeared not to be violated. Posterior probabilities were assessed for estimating the likelihood of each patient belonging to a particular class. Sensitivity analyses were performed to identify recovery trajectories within sub-cohorts based on diagnosis and type of shoulder arthroplasty (ATSA for OA, RTSA for OA vs RTSA for cuff arthropathy or rupture), exploring potential differences in trajectories across diagnosis and arthroplasty types.

**Table 1:** Patient and procedure characteristics of 3,358 total shoulder arthroplasties, stratified by diagnosis and type of shoulder arthroplasty. Values are count (%) unless otherwise specified

Characteristic	All TSAs n = 3,358	ATSA–OA n = 957	RTSA–OA n = 1,207	RTSA–Cuff n = 1,194
Female sex	2,304 (69)	654 (68)	880 (73)	770 (65)
Age, mean (SD)	72 (8.5)	66 (8.6)	74 (7.1)	73 (7.4)
ASA class				
I	307 (9.1)	141 (15)	88 (7.3)	78 (6.5)
II	2,006 (60)	638 (66)	688 (57)	680 (57)
III–IV	1,008 (30)	167 (18)	423 (35)	418 (35)
Missing	37 (1.1)	11 (1.1)	8 (0.7)	18 (1.5)
BMI, mean (SD)	28.3 (5.0)	28.7 (4.9)	28.3 (5.1)	28.0 (4.9)
Missing, n (%)	43 (1.3)	14 (1.5)	10 (0.8)	19 (1.6)
Walch classification				
A1	1,356 (40)	307 (32)	338 (28)	711 (60)
A2	892 (27)	296 (31)	380 (32)	216 (18)
B1	438 (13)	196 (21)	157 (13)	85 (7.1)
B2	327 (9.7)	95 (9.9)	187 (16)	45 (3.8)
B3	112 (3.3)	18 (1.9)	62 (5.1)	32 (2.7)
C	52 (1.5)	5 (0.5)	40 (3.3)	7 (0.6)
Missing	181 (5.4)	40 (4.2)	43 (3.6)	98 (8.2)
Previous surgery on affected shoulder	567 (17)	122 (13)	139 (12)	306 (26)
Missing	13 (0.4)	4 (0.4)	3 (0.2)	6 (0.5)
Smoking	220 (6.6)	77 (8.0)	63 (5.2)	80 (6.7)
Missing	21 (0.6)	2 (0.2)	8 (0.7)	11 (0.9)
Surgical approach				
Anterosuperior/superolateral	569 (17)	4 (0.4)	218 (18)	347 (29)
Deltopectoral	2,738 (82)	938 (98)	971 (80)	829 (69)
Other approach	31 (0.9)	0 (0.0)	18 (1.5)	13 (1.1)
Missing	20 (0.6)	15 (1.6)	0 (0.0)	5 (0.4)
Fixation method				
Cemented	78 (2.3)	56 (5.9)	15 (1.2)	7 (0.6)
Cementless	2,369 (70)	153 (16)	1,100 (91)	1,116 (93)
Hybrid	898 (27)	736 (77)	91 (7.5)	71 (5.9)
Missing	13 (0.4)	12 (1.3)	1 (0.1)	0 (0.0)
Socioeconomic status				
Most deprived	828 (25)	203 (21)	307 (25)	318 (27)
Medium deprived	2,106 (63)	586 (61)	770 (64)	750 (63)
Least deprived	397 (12)	153 (16)	123 (10)	121 (10)
Missing	27 (0.8)	15 (1.6)	7 (0.6)	5 (0.4)
OSS, mean (SD)				
Preoperative	19 (8.0)	21 (7.7)	19 (7.8)	18 (8.2)
Missing, n (%)	343 (10)	113 (12)	89 (7.4)	141 (12)
3-month postop.	29 (11)	29 (11)	30 (11)	27 (11)
Missing, n (%)	530 (16)	142 (15)	197 (16)	191 (16)
12-month postop.	35 (11)	38 (10)	35 (12)	33 (11)
Missing, n (%)	566 (17)	166 (17)	213 (18)	187 (16)

TSA = total shoulder arthroplasty; ATSA = anatomical TSA; RTSA = reversed TSA; OA = osteoarthritis; Cuff = cuff arthropathy or rupture; ASA = American Society of Anesthesiologists; BMI = Body mass index; OSS = Oxford Shoulder Score.

Crude and adjusted multinomial logistic regression analyses were used to investigate associations between potential risk factors and class membership, accounting for classification error, as described by Proust-Lima et al. [13]. These factors were chosen based on expert knowledge, relevant literature, and their availability within the LROI database. Potential risk factors considered included sex, age, diagnosis and type of shoulder arthroplasty, ASA class, BMI, Walch classification, smoking status, previous surgeries on the affected shoulder, and SES. SES was determined by the 4-digit postal code of the patient, and categorized (i.e., most deprived, medium deprived, or least deprived) as described by Bonsel et al. [14]. Briefly, the SES score is calculated for each postal code area with at least 100 inhabitants, based on the mean income per household, the percentage of households with low income, the percentage of unemployed inhabitants, and the percentages of households with low education. Subsequently, the SES scores were categorized into quintiles according to the cumulative z-distribution. Quintile 1 was classified as most deprived, quintiles 2 to 4 as medium deprived, and quintile 5 as least deprived. Surgical approach and fixation method were not considered potential risk factors, as these variables were inherently linked to the type of arthroplasty. The majority of ATSAs for OA (98%) were placed using the deltopectoral approach, and the majority of RTSAs for OA (91%) and RTSAs for cuff arthropathy or rupture (93%) had a cementless fixation (Table 1). A directed acyclic graph (DAG) was created to visualize potential causal relationships between the risk factors, confounders and the outcome (Figure 4, see Appendix). The largest recovery trajectory was considered the reference class. Estimates are presented as odds ratios (OR) with corresponding 95% confidence intervals (CI). Sensitivity analyses for each sub-cohort were not performed in the multinomial logistic regression analyses, as sample sizes were considered too small for meaningful analyses, and similar recovery trajectories with only slightly different class sizes for ATSA for OA, RTSA for OA, and RTSA for cuff arthropathy or rupture were observed.

### Ethics, data sharing, funding, and disclosures

Data was available from the LROI; however, restrictions apply to the availability of this data, which was used under license for the current study. All data was received completely de-identified. The data used in this study is available upon reasonable request, subject to the approval of the LROI. The LROI uses the opt-out system to require informed consent from patients. This study was funded by the LROI. The authors have the following potential conflicts of interest to declare. GH: payment received for meeting hours as member of the Scientific Advisory Board of the LROI, and member of the Data Safety Monitoring Board of the PERSuaDER trial. Complete disclosure of interest forms according to ICMJE are available on the article page, doi: 10.2340/17453674.2025.43085

## Results

Between 2016 and 2022, a total of 13,610 ATSAs and RTSAs for OA, cuff arthropathy, or cuff rupture were registered in the LROI (Figure 1). Of these, 10,208 (75%) cases had PROM data available for only 1 time-point or no PROM data available and were excluded. Among the 3,358 included cases, 1,919 (57%) had PROM data available at all 3 time-points. 343 (10%) cases had a missing preoperative score, 530 (16%) cases had a missing 3-month postoperative score, and 566 (17%) cases had a missing 12-month postoperative score. Of the 3,358 TSAs, 957 were ATSAs for OA, 1,207 were RTSAs for OA, and 1,194 were RTSAs for cuff arthropathy or rupture (Figure 1).

Most TSA patients were female (69%), were ASA II class (60%), had a Walch A1 classification (40%), had not previously undergone surgery on the affected shoulder (83%), were non-smokers (93%), and were operated on with the deltopectoral approach (82%; Table 1). The mean (SD) age was 72 years (8.5), and the mean (SD) BMI was 28.3 (5.0). Table 2 provides an overview of the imputed data.

**Table 2:** Patient and procedure characteristics of all total shoulder arthroplasties, and stratified by class membership

Characteristic	All TSAs		Fast responders		Steady responders		Poor responders	
	n ^a^ (%)	min.–max.^b^	n ^a^ (%)	min.–max.^b^	n ^a^ (%)	min.–max.^b^	n ^a^ (%)	min.–max.^b^
TSAs	3,358		1,999 (59)	1,969–2,041	890 (27)	804–960	469 (14)	427–513
Sex								
Female	2,304 (69)	2,304–2,304	1,310 (65)	1,282–1,337	667 (75)	607–719	327 (70)	298–360
Male	1,054 (31)	1,054–1,054	689 (35)	680–704	223 (25)	197–241	142 (30)	129–153
Age, mean (SD)	71 (8.5)	71–71	72 (8.0)	71–72	72 (9.3)	71–72	71 (8.6)	71–72
ASA class								
I	308 (9.2)	307–310	208 (10)	199–213	76 (8.6)	72–82	25 (5.2)	22–26
II	2,027 (60)	2,020–2,032	1,253 (63)	1,233–1,276	504 (56)	460–537	269 (57)	249–296
III–IV	1,023 (31)	1,019–1,029	538 (27)	521–558	310 (35)	268–342	175 (37)	156–193
BMI, mean (SD)	28 (5.0)	28–28	28 (4.8)	28–28	28 (5.0)	28–28	29 (5.7)	29–29
Walch classification								
A1	1,446 (43)	1,434–1,457	806 (40)	792–817	408 (46)	376–442	232 (50)	206–248
A2	939 (28)	927–947	558 (28)	536–580	251 (28)	213–270	130 (28)	120–149
B1	460 (14)	453–468	289 (15)	277–300	123 (14)	117–132	48 (10)	44–52
B2	340 (10)	335–345	229 (11)	222–234	77 (8.6)	68–88	35 (7.5)	31–41
B3	118 (3.5)	116–121	83 (4.1)	80–86	22 (2.4)	19–25	14 (3.0)	12–16
C	54 (1.6)	52–56	35 (1.7)	30–38	10 (1.1)	8–13	9 (1.9)	7–12
Previous surgery on affected shoulder								
Yes	569 (17)	568–570	309 (16)	301–319	153 (17)	138–169	107 (23)	95–115
No	2,789 (83)	2,788–2,790	1,690 (84)	1,667–1,722	737 (83)	666–791	362 (77)	332–400
Smoking								
Yes	222 (6.6)	221–223	122 (6.1)	118–130	62 (6.9)	54–75	38 (8.2)	30–45
No	3,136 (93)	3,135–3,137	1,876 (94)	1,850–1,914	828 (93)	750–885	431 (92)	397–472
Surgical approach								
Anterosuperior/superolateral	570 (17)	569–571	262 (13)	254–268	136 (15)	115–153	172 (37)	163–186
Deltopectoral	2,757 (82)	2,755–2,758	1,710 (86)	1,680–1,747	751 (84)	686–803	295 (63)	262–324
Other	32 (0.9)	31–33	27 (1.3)	25–29	3 (0.4)	2–4	2 (0.4)	1–3
Fixation method								
Cemented	79 (2.4)	78–82	61 (3.0)	59–65	11 (1.3)	9–15	7 (1.5)	5–8
Cementless	2,372 (71)	2,370–2,375	1,406 (70)	1,382–1,438	613 (69)	538–671	353 (75)	317–395
Hybrid	907 (27)	904–910	532 (27)	525–543	266 (30)	252–278	109 (23)	100–115
Socioeconomic status								
Most deprived	836 (25)	833–839	459 (23)	445–473	240 (27)	213–270	136 (29)	119–150
Medium deprived	2,122 (63)	2,117–2,125	1,299 (65)	1,282–1,328	551 (62)	500–586	272 (58)	253–297
Least deprived	400 (12)	397–402	240 (12)	234–247	100 (11)	91–107	61 (13)	55–67
Diagnosis and type of arthroplasty								
ATSA for OA	957 (28)	957–957	600 (30)	595–609	259 (29)	249–270	98 (21)	90–102
RTSA for OA	1,207 (36)	1,207–1,207	757 (38)	738–776	282 (32)	245–319	168 (36)	150–186
RTSA for cuff	1,194 (36)	1,194–1,194	642 (32)	632–656	349 (39)	310–371	204 (43)	185–228

For Abbreviations, see Table 1.

Estimates are pooled across the 10 imputed datasets.

a Pooled cases are rounded to the nearest number.

b Lowest and highest values across the 10 imputed datasets.

### Model selection

The pooled relative fit statistics showed a decrease from the 1-class to the 4-class model, suggesting that a higher number of classes provided a better fit (Table 3). The 4-class model revealed minor variations in recovery trajectories, and was considered less clinically relevant. Therefore, the model with 3 classes was selected as the final model. The pooled mean (SD) posterior probabilities of class membership in the 3-class model were 0.89 (0.14) for class 1, 0.76 (0.15) for class 2, and 0.84 (0.17) for class 3, indicating satisfactory model performance (Table 4).

**Table 3:** Pooled model fit statistics

Model	Log likelihood	AIC	BIC	Adjusted BIC	Class (%)			
					1	2	3	4
All TSAs								
1-class	–37,130	74,269	74,300	74,284	100			
2-class	–36,583	73,184	73,240	73,211	80	20		
3-class	–36,373	72,772	72,852	72,811	59	27	14	
4-class	–36,289	72,611	72,715	72,661	55	32	8.9	4.5
ATSA–OA								
1-class	–10,478	20,970	20,993	20,978	100			
2-class	–10,334	20,686	20,729	20,701	82	18		
3-class	–10,236	20,499	20,562	20,521	61	29	10	
4-class	–10,228	20,490	20,573	20,519	52	29	12	7.1
RTSA–OA								
1-class	–13,377	26,763	26,789	26,773	100			
2-class	–13,138	26,295	26,341	26,312	80	20		
3-class	–13,068	26,161	26,228	26,186	63	23	14	
4-class	–13,066	26,165	26,252	26,198	60	25	14	1.6
RTSA–Cuff								
1-class	–13,208	26,425	26,451	26,435	100			
2-class	–13,034	26,086	26,132	26,103	77	23		
3-class	–12,977	25,981	26,047	26,005	55	28	17	
4-class	–12,935	25,904	25,990	25,936	48	35	12	4.7

AIC = Akaike information criterion; BIC = Bayesian information criterion.

For Abbreviations, also see Table 1.

**Table 4:** Pooled posterior probabilities of class membership in the 3-class model. Values are mean (standard deviation)

Class	All TSAs n = 3,358	ATSA–OA n = 957	RTSA–OA n = 1,207	RTSA–Cuff n = 1,194
Class 1	0.89 (0.14)	0.91 (0.13)	0.90 (0.13)	0.86 (0.15)
Class 2	0.76 (0.15)	0.81 (0.15)	0.75 (0.16)	0.73 (0.15)
Class 3	0.84 (0.17)	0.86 (0.16)	0.85 (0.17)	0.82 (0.17)

For Abbreviations, see Table 1.

Sensitivity analyses showed that the 3-class models were also the most appropriate for ATSA for OA, RTSA for OA, and RTSA for cuff arthropathy or rupture (Table 3). The pooled mean (SD) posterior probabilities of class membership were 0.91 (0.13) for class 1, 0.81 (0.15) for class 2, and 0.86 (0.16) for class 3 among ATSAs for OA; 0.90 (0.13) for class 1, 0.75 (0.16) for class 2, and 0.85 (0.17) for class 3 among RTSAs for OA; and 0.86 (0.15) for class 1, 0.73 (0.15) for class 2, and 0.82 (0.17) for class 3 among RTSAs for cuff arthropathy or rupture (Table 4).

### Recovery trajectories

**Figure 2:**
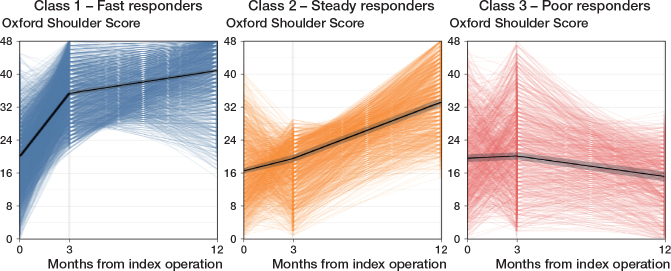
Recovery trajectories based on Oxford Shoulder Scores, stratified by class membership (Class 1: n = 1,999; Class 2: n = 890; Class 3: n = 469). Pooled mean trajectories with corresponding 95% CIs are shown in black, and individual trajectories are displayed in color. Individuals were classified into classes according to their highest probability of class membership.

The final model showed 3 different recovery trajectories after TSA, which were labelled as “Fast responders,” “Steady responders,” and “Poor responders” (Figure 2).

The “Fast responders” was the largest class, including 1,999 (59%) TSAs. This class had a pooled estimated preoperative OSS of 20 (CI 20–21). The pooled estimated OSS increased sharply to 35 (CI 35–36) at 3 months postoperatively, followed by a further rise to 41 (CI 40–41) at 12 months postoperatively, which indicates better physical function and reduced pain.

The second class was the “Steady responders,” consisting of 890 (27%) TSAs. The pooled estimated preoperative OSS of this class was slightly lower (17, CI 16–17) than that of the “Fast responders.” The pooled estimated OSS had a steady increase to 20 (CI 19–20) at 3 months postoperatively, and to 33 (CI 32–34) at 12 months postoperatively, indicating improved physical function and pain.

The smallest class, comprising 469 (14%) TSAs, was labelled “Poor responders.” This class had a comparable pooled estimated OSS of 20 (CI 19–21) preoperatively to the “Fast responders.” No improvements were observed at 3 months postoperatively (20, CI 19–21). The pooled estimated OSS declined to 15 (CI 14–16) at 12 months postoperatively, indicating worse physical function and increased pain.

**Figure 3:**
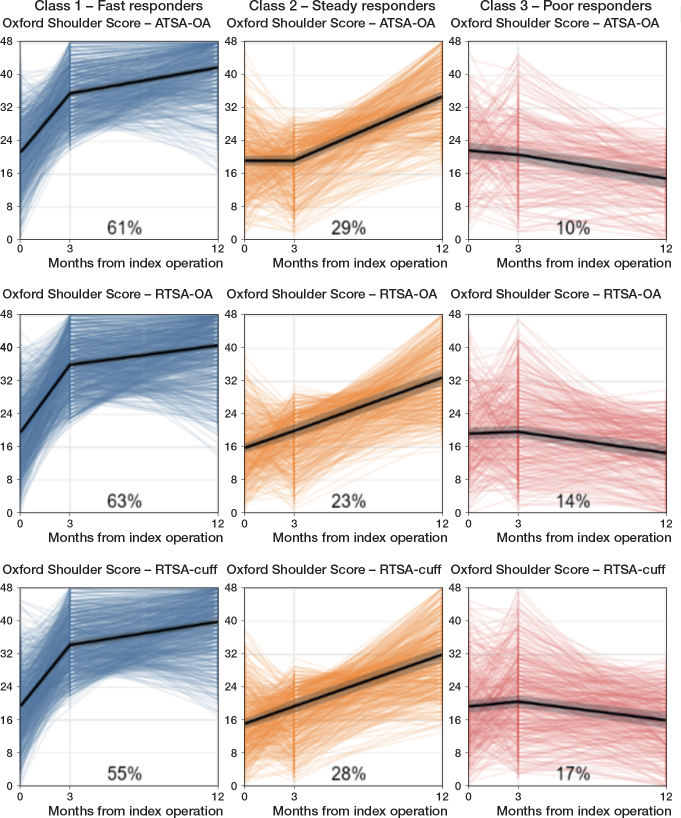
Recovery trajectories based on Oxford Shoulder Scores, stratified by class membership, and diagnosis and arthroplasty type. Pooled mean trajectories with corresponding 95% CIs are shown in black, and individual trajectories are displayed in color. Individuals were classified into classes according to their highest probability of class membership. Class sizes in percentages are shown for each sub-cohort. ATSA = total anatomical shoulder arthroplasty; RTSA = total reversed shoulder arthroplasty; OA = osteoarthritis; Cuff = cuff arthropathy or rupture.

Sensitivity analyses revealed similar recovery trajectories with slightly different class sizes for ATSA for OA, RTSA for OA, and RTSA for cuff arthropathy or rupture (Figure 3).

**Figure 4:**
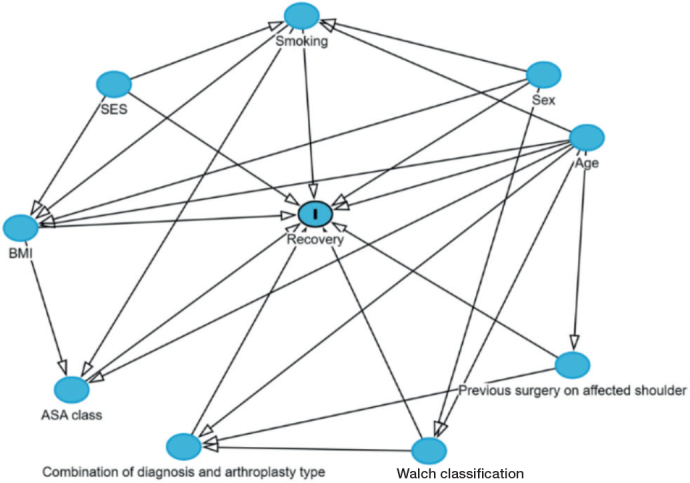
Directed acyclic graph (DAG) illustrating the potential relationships between risk factors and recovery trajectories following total shoulder arthroplasty. Confounders were chosen according to this DAG: ASA class was adjusted for age, BMI, and smoking; BMI was adjusted for sex, age, smoking, and SES; Walch classification was adjusted for sex and age; previous surgeries on affected shoulder was adjusted for age; smoking was adjusted for sex, age, and SES; and diagnosis and arthroplasty type was adjusted for age, previous surgeries on affected shoulder, and Walch classification. No adjustments were made for sex, age, and SES.

### Potential risk factors and class membership

Pooled adjusted multinomial regression analyses showed that factors statistically significantly associated with class membership in the “Steady responders” vs the “Fast responders” were female vs male sex (OR 2.0, CI 1.5–2.7), ASA III–IV vs ASA I (OR 1.9, CI 1.2–3.1), Walch A1 vs B2 (OR 1.6, CI 1.1–2.5), and most vs medium socioeconomic deprivation (OR 1.4, CI 1.1–1.9). Factors statistically significantly associated with class membership in the “Poor responders” vs the “Fast responders” included ASA II vs ASA I (OR 2.0, CI 1.1–3.6), ASA III–IV vs ASA I (OR 3.0, CI 1.6–5.5), Walch A1 vs B2 (OR 2.1, CI 1.3–3.3), previous shoulder surgeries (OR 1.8, CI 1.3–2.4), most vs medium socioeconomic deprivation (OR 1.5, CI 1.2–2.0), RTSA for OA vs ATSA for OA (OR 1.8, CI 1.2–2.7), and RTSA for cuff arthropathy or rupture vs ATSA for OA (OR 2.3, CI 1.5–3.4; Table 5).

**Table 5:** Crude and adjusted multinomial logistic regression analyses of potential risk factors and class membership

Factor	“Steady responders” vs “Fast responders”		“Poor responders” vs “Fast responders”	
	Crude OR (CI)	Adjusted ^a^ OR (CI)	Crude OR (CI)	Adjusted ^a^ OR (CI)
Females (ref. males)	2.0 (1.5–2.7)	2.0 (1.5–2.7)	1.3 (1.0–1.6)	1.3 (1.0–1.6)
Age	1.0 (1.0–1.0)	1.0 (1.0–1.0)	1.0 (1.0–1.0)	1.0 (1.0–1.0)
ASA class (ref. ASA I)				
II	1.1 (0.7–1.7)	1.1 (0.7–1.7)	2.1 (1.2–3.7)	2.0 (1.1–3.6)
III–IV	1.9 (1.1–3.0)	1.9 (1.2–3.1)	3.2 (1.8–5.8)	3.0 (1.6–5.5)
Body mass index	1.0 (1.0–1.0)	1.0 (1.0–1.0)	1.1 (1.0–1.1)	1.1 (1.0–1.1)
Walch classification (ref. B2)				
A1	1.7 (1.1–2.6)	1.6 (1.1–2.5)	2.1 (1.3–3.3)	2.1 (1.3–3.3)
A2	1.5 (0.9–2.3)	1.4 (0.9–2.1)	1.6 (1.0–2.6)	1.6 (1.0–2.6)
B1	1.3 (0.8–2.2)	1.3 (0.7–2.1)	1.0 (0.6–1.7)	1.0 (0.6–1.8)
B3	0.7 (0.3–1.7)	0.8 (0.3–1.7)	1.1 (0.5–2.5)	1.1 (0.5–2.6)
C	0.9 (0.3–3.0)	0.9 (0.3–3.1)	2.0 (0.7–5.6)	2.0 (0.7–5.7)
Previous surgeries on affected shoulder (ref. no previous surgeries)	1.2 (0.9–1.6)	1.2 (0.9–1.6)	1.8 (1.3–2.4)	1.8 (1.3–2.4)
Smoking (ref. no smoking)	1.3 (0.8–2.0)	1.4 (0.8–2.2)	1.3 (0.8–2.3)	1.3 (0.8–2.3)
Socioeconomic status (ref. medium deprived)				
Most deprived	1.4 (1.1–1.9)	1.4 (1.1–1.9)	1.5 (1.2–2.0)	1.5 (1.2–2.0)
Least deprived	1.1 (0.7–1.7)	1.1 (0.7–1.7)	1.3 (0.9–1.8)	1.3 (0.9–1.8)
Diagnosis and arthroplasty type (ref. ATSA–OA)				
RTSA–OA	0.8 (0.6–1.1)	0.8 (0.6–1.1)	1.5 (1.1–2.2)	1.8 (1.2–2.7)
RTSA–Cuff	1.5 (1.1–2.0)	1.4 (1.0–1.9)	2.3 (1.6–3.3)	2.3 (1.5–3.4)

Estimates are pooled across the 10 imputed datasets.

a Confounders were chosen according to the directed acyclic graph (Figure 4, see Appendix).

OR = odds ratio; CI = 95% confidence interval. For Abbreviations, also see Table 1.

## Discussion

We aimed to identify recovery trajectories after TSA based on the Oxford Shoulder Score (OSS) along with their potential risk factors. A total of 3 recovery trajectories were identified after TSA, which we labelled as “Fast responders,” “Steady responders,” and “Poor responders.” Both “Fast responders” and “Steady responders” showed improved physical function and reduced pain 12 months postoperatively, comprising most patients (86%). In contrast, “Poor responders” experienced slightly worsened physical function and increased pain. “Steady responders” and “Poor responders” were more likely to have higher ASA scores, a Walch A1 classification, and greater socioeconomic deprivation than “Fast responders”, compared with the reference categories. Moreover, “Steady responders” were more likely to be female, while “Poor responders” were more likely to have previous shoulder surgeries than “Fast responders.”

Similar recovery trajectories were observed following ATSA for OA, RTSA for OA, and RTSA for cuff arthropathy or rupture, although the class sizes differed slightly among these groups. Compared with patients with ATSA for OA, patients with RTSA for OA and for cuff arthropathy or rupture were more likely to be “Poor responders” than “Fast responders.” These differences may be explained by differences in patient characteristics between the sub-cohorts. In our study, patients with RTSA for OA, cuff arthropathy, or cuff rupture were older and had higher ASA scores than those with ATSA for OA. Moreover, patients with RTSA for cuff arthropathy or rupture had undergone previous shoulder surgeries more often than those with ATSA or RTSA for OA. Although we performed adjusted multinomial logistic regression analyses based on a DAG to minimize confounding, residual confounding is likely still present. The use of registry data, which is collected as part of the usual care process, is limited by the number of variables collected. Possible risk factors for recovery, such as preoperative mental health status and diabetes, could not be included in this study [15-17]. Therefore, the findings regarding diagnosis and arthroplasty type should be interpreted carefully, as unmeasured factors may have influenced the associations between these variables and class membership.

A minority of patients (14%) did not experience improvement after TSA, leading to their classification as “Poor responders.” This may include patients with complications and/or revisions occurring more than 1 year postoperatively. This proportion is consistent with other studies, which report that 9% to 16% of patients experience no improvement or continue to suffer from persistent pain 2 years after ATSA or RTSA for OA, cuff arthropathy, or cuff rupture [3,18,19].

Only 1 previous study has identified recovery trajectories after TSA, finding 3 different trajectories: “High performers,” “Steady progressors,” and “Resistant responders” [9]. The “High performers” and “Steady progressors” seem to be comparable to the “Fast responders” and “Steady responders” in our study. However, while “Resistant responders” showed small improvement, our “Poor responders” experienced slightly worsened physical function and increased pain. This difference may be partly explained by differences in methodological aspects. The previous study was conducted at a single center and included only complete cases, resulting in a smaller sample size compared with our national registry study, covering all Dutch hospitals performing TSA and collecting OSS. Moreover, the previous study used the American Shoulder and Elbow Surgeons Standardized Shoulder Assessment, measured at 5 time-points up to 2 years postoperatively, whereas we used the OSS, measured at 3 time-points up to 1 year postoperatively. In contrast to our results, the study also found differences in the smallest recovery trajectories after ATSA and RTSA, with “Late responders” observed after ATSA and “Late regressors” after RTSA. Their results were stratified by type of shoulder arthroplasty, while we stratified our results by both diagnosis and type of shoulder arthroplasty. Furthermore, 8% of the ATSAs and 13% of the RTSAs were performed for diagnoses other than OA, cuff arthropathy, or cuff rupture [9].

Various studies have explored the relationship between patient and procedure factors and patient-reported outcomes following ATSA or RTSA [15,16,18-22]. Most studies, including ours, found that a history of shoulder surgeries was associated with worse patient-reported outcomes [15,18-21]. Some studies identified comorbidity as a risk factor for worse patient-reported outcomes, which may be reflected by our association of higher ASA scores with suboptimal recovery [16,18,19]. Although both our and another study found an association between sex and patient-reported outcomes [15], other studies did not observe this link [18,19,21,22], potentially due to differences in study design, sample sizes, and methodology. In contrast to our findings, smoking was also identified as risk factor in previous studies [15,20,22]. However, confidence intervals were wide in our study, including for smoking, resulting in uncertainty around the effect estimates.

### Limitations

PROM response rate after TSA is relatively low. Of the 13,610 TSAs registered for OA, cuff arthropathy, or cuff rupture between 2016 and 2022, 3,358 (25%) TSA patients had at least 2 out of 3 PROM scores available and were therefore included in this study. This response rate is well below the 60% threshold proposed by the International Society of Arthroplasty Registries as an acceptable response rate, suggesting that our results may be affected by non-responder bias [23]. PROM non-responders were more frequently treated in the early years of the study (i.e., 2016–2017) and were therefore less often operated on using the deltopectoral approach than responders. No other differences were observed in patient and procedure characteristics between non-responders and responders. Moreover, the response rate of PROMs varies widely among Dutch hospitals, with some hospitals not collecting any PROM data, which may negatively impact the generalizability of this study [10]. However, no associations were found between hospital-level PROM response rates and patient characteristics such as age, sex, BMI, and ASA class in Australia, where PROM response rates range from 4.5% to 82% for preoperative scores and from 3.8% to 69% for 6-month postoperative scores [24]. This may suggest that higher hospital-level PROM response rates may not necessarily result in a more representative sample [24]. Lastly, the OSS may be subject to ceiling effects 6 to 12 months postoperatively [25]. However, 11% of patients across the 10 imputed datasets achieved the maximum OSS score at 12 months postoperatively in our study, which is below the commonly used 15% threshold [26].

### Conclusion

3 recovery trajectories were identified following TSA for OA, cuff arthropathy, or cuff rupture, which were labelled as “Fast responders,” “Steady responders,” and “Poor responders.” Both ATSA and RTSA seem to follow similar recovery trajectories, despite the differences in diagnoses and arthroplasty type. “Steady responders” and “Poor responders” were more likely to have higher ASA scores, a Walch A1 vs B2 classification, and greater vs medium socioeconomic deprivation than “Fast responders.” Moreover, “Steady responders” were more likely to be female, while “Poor responders” were more likely to have had previous shoulder surgeries than “Fast responders.”

*In perspective,* the recovery trajectories and their associated risk factors may provide valuable guidance for orthopedic surgeons in counseling patients undergoing TSA.

## Notes

MHWvV, LNvS, CPJV, BWS, GH: concept and design. MHWvV, LNvS, CPJV, BWS, GH: data analysis and interpretation. MHWvV: manuscript preparation. MHWvV, LNvS, CPJV, BWS, GH: manuscript editing. MHWvV, LNvS, CPJV, BWS, GH: manuscript review. MHWvV, LNvS, CPJV, BWS, GH: final approval of the version submitted.

Handling co-editors: Jeppe Vejlgaard Rasmussen and Philippe Wagner

*Acta* thanks Erica Domeij-Arverud and Randi Margrete Hole for help with peer review of this manuscript.
